# Female genital tuberculosis presenting as a protruding anterior vaginal wall mass: a case report

**DOI:** 10.3389/fmed.2024.1502969

**Published:** 2025-01-22

**Authors:** Mequanint Melesse Bicha, Tewodros Zenabu Kebede, Ayalew Lingerih Arefeaynie, Eden Woldegerima Meressa

**Affiliations:** ^1^Department of Obstetrics and Gynecology, School of Medicine, University of Gondar, Gondar, Ethiopia; ^2^Department of Medical Laboratory, School of Biomedical Sciences, University of Gondar, Gondar, Ethiopia; ^3^Department of Medical Biotechnology, Institute of Biotechnology, University of Gondar, Gondar, Ethiopia

**Keywords:** female genital tract tuberculosis, vagina, tuberculosis, vaginal wall mass, case report

## Abstract

Although pulmonary tuberculosis is a common infectious disease, especially in low-and middle-income countries, female genital tract tuberculosis (TB) is rarely reported. Female genital TB might be asymptomatic or manifest clinically in an unusual way, making an early diagnosis challenging. The most often affected regions of the genital system are the fallopian tubes and endometrium. Menstrual abnormalities, infertility, and chronic pelvic pain are frequent presenting symptoms. Rare reports of vulvar and vaginal TB exist. This case report features a 35-year-old woman who had a bulging tumor in her vagina for a year before being identified with anterior vaginal wall TB, treated with anti-tuberculosis medication, and made improvements.

## Introduction

Tuberculosis (TB) is a communicable disease that is a major cause of morbidity and mortality. Its etiologic agent is a bacterium called *Mycobacterium tuberculosis* (1). The disease typically affects the lungs (pulmonary TB) but can affect other sites as well (2). A survey done in the national adult population in Ethiopia estimated that the prevalence of smear-positive TB was 108/100000 and that of bacteriologically confirmed TB was 277/100000 (3). Morgagni documented the first instance of female genital tuberculosis in 1744, following a post-mortem examination of a 20-year-old lady who died of tuberculosis and whose uterus and tubes were found to be packed with caseous contents (4, 5).

Female genital tuberculosis (FGTB) is a kind of extrapulmonary tuberculosis (EPTB) that affects the female reproductive organs, most commonly the fallopian tubes (90%), ovaries (10–30%), and endometrium (50%). The highest incidence of genital tuberculosis occurs in child-bearing age women. Because it is detected in approximately 10% of individuals with pulmonary tuberculosis, one may expect a high incidence of pelvic tuberculosis in areas where the incidence of pulmonary tuberculosis is high (6).

FGTB can present with chronic pelvic inflammatory disease, menstrual abnormalities, and infertility (7). The actual number of FGTB incidences cannot be estimated accurately, as it is often asymptomatic, and only 50% of cases are diagnosed without surgery (8). Vaginal tuberculosis is extremely rare (9) and may present as a differential of vaginal cancer (10). Vaginal tuberculosis was also found as a multifocal mass in the area of the vaginal introitus, with the main lesion reaching a diameter of 3 cm (11). This case report described an uncommon symptom of genital TB in the vagina, which has been rarely recorded in the literature.

## Case presentation

**Figure 1:**
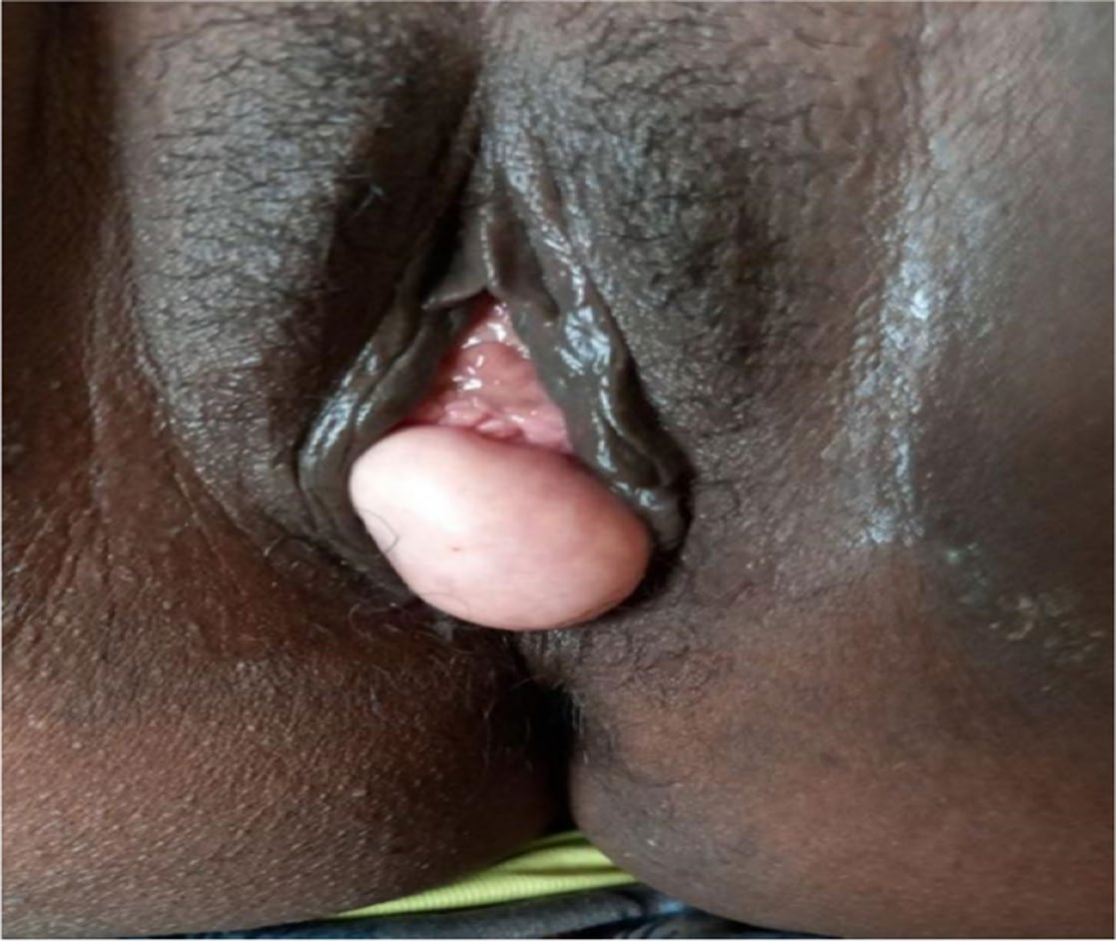
Protruding cystic mass through the vagina arising from the mid-anterior vaginal wall.

**Figure 2:**
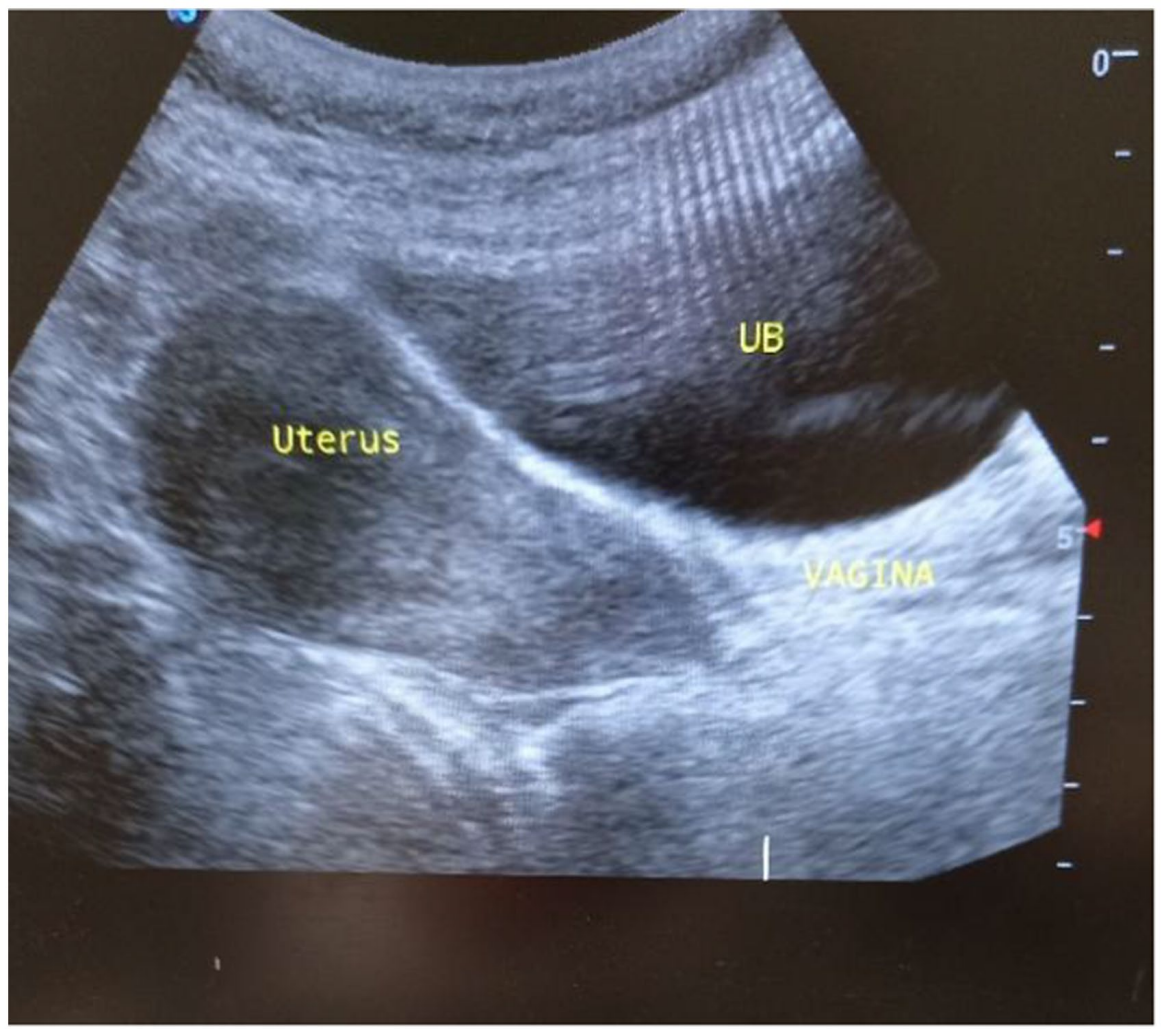
Pelvic ultrasound showing a sagittal view of a normal uterine and cervical outline, UB, Urinary bladder.

**Figure 3:**
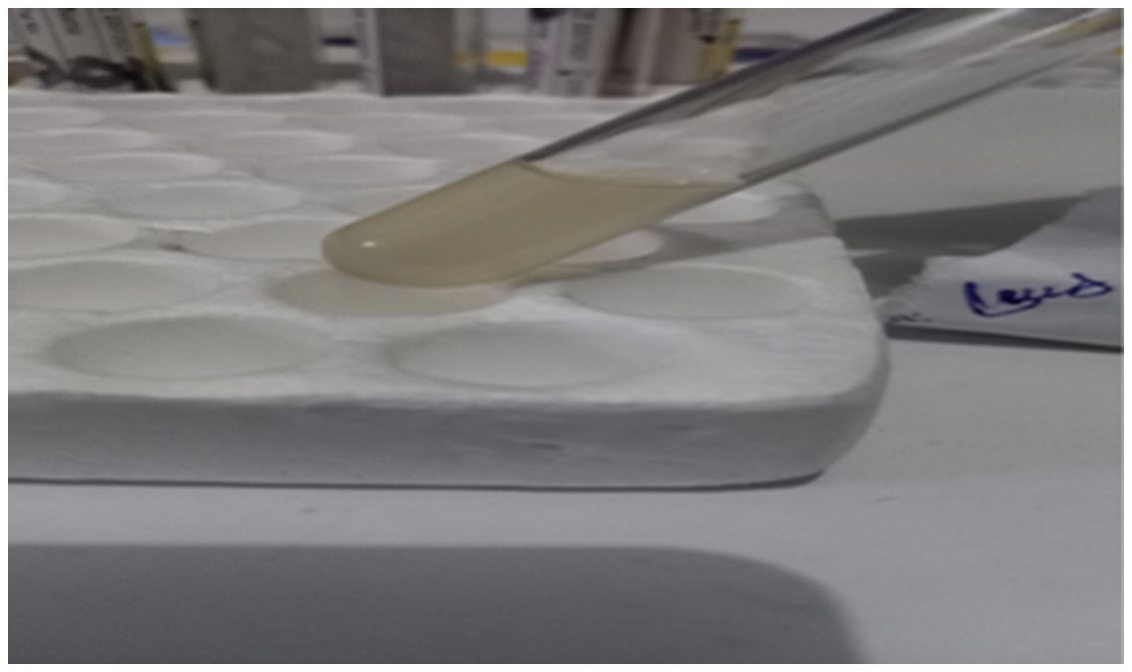
Aspirated fluid from the anterior vaginal wall mass.

**Figure 4:**
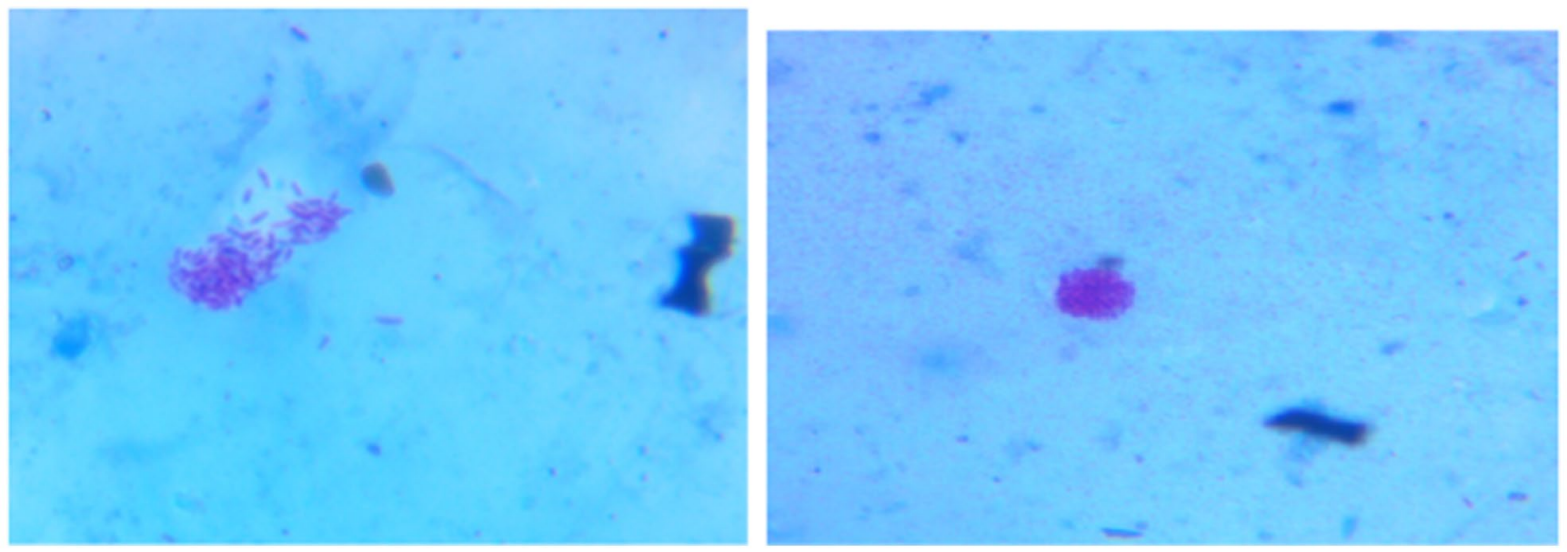
Acid-fast bacilli seen under the microscope from the aspirate fluid.

**Figure 5:**
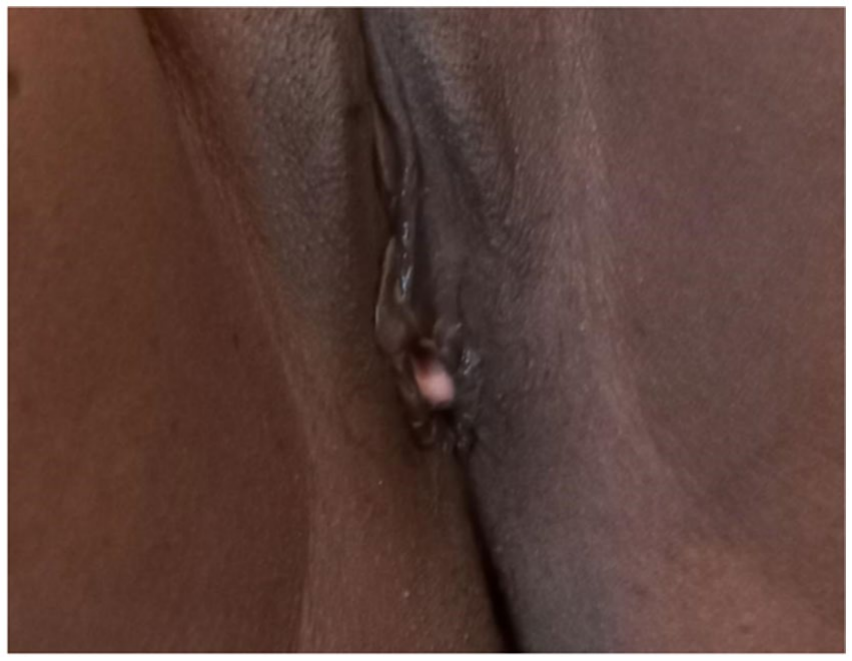
Protruded cystic mass disappeared after anti-TB treatment (6th month).

A 35-year-old Para Five woman presented with a 1-year history of a progressively increasing bulging mass per vagina which was initially small but increased in size progressively. She also reported loss of appetite and a 30% weight loss. She had severe dyspareunia and discomfort during vaginal intercourse. She had five children, the youngest of whom was 4 years old. She delivered all of her children vaginally. After the last child, she was using Depo-Provera injection as a contraceptive method. She had no history of past TB treatment and no contact with a known TB patient. For these complaints, she visited a health center and was referred for further evaluation after she was told that her diagnosis was uterovaginal prolapse. She came to our hospital with these complaints, her history was taken, and she was examined. Her vital signs were all in the normal range, but she appeared emaciated; her weight was 35 kg, and her BMI was 13 kg/m^2^. On examination of the external genitalia, there was a 5 by 4 cm pink smooth protruded mass outside the introitus (Figure 1) which had a cystic consistency, was non-tender, and emerged from the mid-anterior vaginal wall. The urethral opening, the distal and proximal anterior vaginal walls, the lateral and posterior vaginal walls, the vaginal fornixes, and the cervix are all normal. Pelvic ultrasound revealed a normal size and outline of the urinary bladder, uterus, and adnexa (Figure 2). On laboratory evaluation, the erythrocyte sedimentation rate (ESR) was 100 mm/h, and a complete blood count showed lymphocytosis. The chest x-ray showed normal findings. Fine needle aspiration was done on the cystic mass (Figure 3) and using Ziehl Neelson Staining, microscopy showed Acid-Fast Bacilli (Figure 4). The sample was sent for culture, and tuberculosis was confirmed. The patient was given standard daily anti-tuberculosis therapy (isoniazid, rifampicin, ethambutol, and pyrazinamide) for the first 2 months and continued daily therapy with isoniazid and rifampicin for the next 4 months, and the anti-TB was given for a total of 6 months. She was counseled on drug adherence, put on a high-protein diet, and had an optimal follow-up in the hospital. After she completed the treatment, she was examined and the bulging mass per vagina disappeared (Figure 5), her BMI was corrected, and her overall wellbeing improved a lot.

## Discussion and conclusion

Genitourinary TB was reported to be responsible for 27.1 percent of cases of EPTB with genital TB being seen in 9 percent of cases (12). However, the exact incidence of FGTB is not known due to underreporting, asymptomatic cases, vague symptoms, and the lack of reliable and highly sensitive diagnostics (13, 14). The diagnosis of FGTB is made by detecting acid-fast bacilli under microscopy or culture on endometrial biopsy, or by detecting epithelioid granuloma during biopsy. The polymerase chain reaction is insufficient to make a diagnosis because of the higher rate of false positives. Laparoscopy and hysteroscopy can detect genital TB based on numerous findings (13). In our case, acid-fast bacilli were seen on a sample taken from the vaginal mass under microscopy, and tuberculosis was confirmed with culture.

Genital tract TB has been associated with up to 21% of infertility cases in low-and middle-income countries, due to tubal obstruction or adhesions in the uterine cavity (15, 16) and is even higher in patients with tubal factor infertility (17). Although the symptoms of genital TB are often absent, patients can present with infertility, pelvic/abdominal pain, or menstrual disturbances (18). Tuberculosis in the genital tract is usually secondary to tuberculosis elsewhere, most commonly in the lungs, but also in the kidneys, gastrointestinal tract, bones, or joints. (19). In our case, the chest x-ray was normal, and the patient did not have clinical evidence of tuberculosis in the internal female genital tract. Primary vaginal wall tuberculosis is highly likely.

Tuberculosis affecting the vagina and vulva is uncommon (20, 21) and is usually an extension from the endometrium or cervix, or very rarely primary due to transmission from an infected partner’s semen (22). Our case did not have menstrual abnormalities, infertility, or cervicitis. Her partner was not having tuberculosis. A hypertrophic ulcer or growth on the vulva or vagina may necessitate a biopsy and histological demonstration of granuloma, as well as the exclusion of cancer and other diseases such as syphilis and lymphogranuloma venereum (13, 14, 23). In our case, there was neither an ulcer nor hypertrophic mass, but it was just a cystic and soft anterior vaginal wall swelling protruding through the introitus. A giant vulval tumor has also been reported in FGTB (24). Even vesicovaginal and rectovaginal fistulas have also been reported (25). In this case, the vesicovaginal septum was normal before, during, and after treatment, and the mass disappeared with no sequelae.

Genital TB should be treated with anti-tuberculous therapy consisting of rifampin, isoniazid, pyrazinamide, ethambutol (RIPE) for 2 months followed by rifampin and isoniazid for 4 months (26). In this case, the standard anti-TB treatment was administered with proper follow-up for 6 months, and the patient improved.

In conclusion, it is always better to have a high index of suspicion in any mass arising from the female genital tract, particularly in areas where tuberculosis is rampant. Vaginal tuberculosis could be one of the differential diagnoses in women presenting with vaginal mass.

## Data Availability

The original contributions presented in the study are included in the article/supplementary material, further inquiries can be directed to the corresponding author.

## References

[1] Chakaya J Khan M Ntoumi F Aklillu E Fatima R Mwaba P. Global tuberculosis report 2020–reflections on the global TB burden, treatment and prevention efforts. Int J Infect Dis. (2021) 113:S7–S12. doi: 10.1016/j.ijid.2021.02.107, PMID: 33716195 PMC8433257

[2] World Health Organization. Global tuberculosis report 2021. Geneva: World Health Organization (2022).

[3] Kebede A Alebachew Z Tsegaye F Lemma E Abebe A Agonafir M. The first population-based national tuberculosis prevalence survey in Ethiopia, 2010-2011. Int J Tuberc Lung Dis. (2014) 18:635–9. doi: 10.5588/ijtld.13.0417, PMID: 24903931

[4] Sharma JB Sharma E Sharma S Dharmendra S. Female genital tuberculosis: revisited. Indian J Med Res. (2018) 148:S71–83. doi: 10.4103/ijmr.IJMR_648_18, PMID: 30964083 PMC6469382

[5] Mustafa M Amin B Gayas S. Female genital tuberculosis in infertile women. Arch Anesthesiol Crit Care. (2022) 8:226–9. doi: 10.18502/aacc.v8i3.9615, PMID: 39712361

[6] Rao K. Textbook of tuberculosis. Ghaziabad, India: Vikas Publishing House (1981).

[7] Bapna N Swarankar M Kotia N. Genital tuberculosis and its consequences on subsequent fertility. J Obstet Gynaecol India. (2005) 55:534–7.

[8] Abebe M Lakew M Kidane D Lakew Z Kiros K Harboe M. Female genital tuberculosis in Ethiopia. Int J Gynecol Obstet. (2004) 84:241–6. doi: 10.1016/j.ijgo.2003.11.002, PMID: 15001372

[9] Nogales-Ortiz F Tarancón I Nogales FF Jr. The pathology of female genital tuberculosis. A 31-year study of 1436 cases. Obstet Gynecol. (1979) 53:422–8. PMID: 440643

[10] Carton I Balès D Bargain A Lemoine PLP. Vaginal tuberculosis as differential diagnosis of cancer: a case report. J Gynecol Obstet Hum Reprod. (2021) 50:101873. doi: 10.1016/j.jogoh.2020.101873, PMID: 32693050

[11] Alhakeem M Schneider A. Genital tuberculosis–a rare cause for vulvovaginal discharge and swelling. J Microbiol Infect Dis. (2013) 3:141–3. doi: 10.5799/ahinjs.02.2013.03.0097

[12] Golden MP Vikram HR. Extrapulmonary tuberculosis: an overview. Am Fam Physician. (2005) 72:1761–8. PMID: 16300038

[13] Sharma JB. Current diagnosis and management of female genital tuberculosis. J Obstet Gynaecol India. (2015) 65:362–71. doi: 10.1007/s13224-015-0780-z, PMID: 26663993 PMC4666212

[14] Neonakis IK Spandidos DA Petinaki E. Female genital tuberculosis: a review. Scand J Infect Dis. (2011) 43:564–72. doi: 10.3109/00365548.2011.568523, PMID: 21438789

[15] Aliyu MH Aliyu SH Salihu HM. Female genital tuberculosis: a global review. Int J Fertil Womens Med. (2004) 49:123–36. PMID: 15303314

[16] Mondal SK Dutta T. A ten year clinicopathological study of female genital tuberculosis and impact on fertility. J Nepal Med Assoc. (2008) 48:202. doi: 10.31729/jnma.202 19529059

[17] Fowler ML Mahalingaiah S. Case report of pelvic tuberculosis resulting in Asherman’s syndrome and infertility. Fertil Res Pract. (2019) 5:1–3. doi: 10.1186/s40738-019-0061-0 31388435 PMC6670196

[18] Sharma JB Roy KK Pushparaj M Gupta N Jain SK Malhotra N. Genital tuberculosis: an important cause of Asherman’s syndrome in India. Arch Gynecol Obstet. (2008) 277:37–41. doi: 10.1007/s00404-007-0419-0, PMID: 17653564

[19] Varma T. Genital tuberculosis and subsequent fertility. Int J Gynecol Obstet. (1991) 35:1–11. doi: 10.1016/0020-7292(91)90056-B, PMID: 1680069

[20] Akhlaghi F Hamedi A. Postmenopausal tuberculosis of the cervix, vagina and vulva. Int J Gynaecol Obstet. (2004) 3:e6. doi: 10.5580/15e6, PMID: 440643

[21] Manoj K Soma M Ajay L Ashish A Rakesh S Paliwal R. Tubercular sinus of labia majora: rare case report. Infect Dis Obstet Gynecol. (2008) 2008:1–3. doi: 10.1155/2008/817515, PMID: 18301724 PMC2248239

[22] Parikh FR Nadkarni SG Kamat SA Naik N Soonawala SB Parikh RM. Genital tuberculosis—a major pelvic factor causing infertility in Indian women. Fertil Steril. (1997) 67:497–500. doi: 10.1016/S0015-0282(97)80076-3, PMID: 9091337

[23] Tiwari P Pal DK Moulik D Choudhury MK. Hypertrophic tuberculosis of vulva--a rare presentation of tuberculosis. Indian J Tuberc. (2010) 57:95–7. PMID: 21114177

[24] Kumar S Kameshwarachari P Ray R. Giant vulval tumor due to tuberculosis. Int J Gynaecol Obstet. (2010) 110:69–70. doi: 10.1016/j.ijgo.2010.02.010, PMID: 20409547

[25] Sharma J Sharma K Sarin U. Tuberculosis: a rare cause of rectovaginal fistula in a young girl. J Obstet Gynaecol India. (2001) 51:176.

[26] Buppasiri P Temtanakitpaisan T Somboonporn W. Tuberculosis at vulva and vagina. J Med Assoc Thail. (2010) 93:613–5. PMID: 20524449

